# A first-in-human, open-label Phase 1b and a randomised, double-blind Phase 2a clinical trial in recent-onset type 1 diabetes with AG019 as monotherapy and in combination with teplizumab

**DOI:** 10.1007/s00125-023-06014-2

**Published:** 2023-10-02

**Authors:** Chantal Mathieu, Alice Wiedeman, Karen Cerosaletti, S. Alice Long, Elisavet Serti, Laura Cooney, Joan Vermeiren, Silvia Caluwaerts, Karolien Van Huynegem, Lothar Steidler, Sven Blomme, Pieter Rottiers, Gerald T. Nepom, Kevan C. Herold, on behalf of the AG019-T1D-101 Trial Investigators

**Affiliations:** 1 Clinical and Experimental Endocrinology, University Hospital of Leuven, Leuven, Belgium; 2 Center for Translational Immunology, Benaroya Research Institute, Seattle, WA USA; 3 Immune Tolerance Network, Bethesda, MD USA; 4 Precigen ActoBio, Ghent, Belgium; 5 Department of Immunology and Internal Medicine, Yale University, New Haven, CT USA

**Keywords:** AG019, Anti-CD3 monoclonal antibody, Antigen-specific immunotherapy, IL-10, *Lactococcus lactis* bacteria, Oral tolerance, Proinsulin, Teplizumab, Type 1 diabetes

## Abstract

**Aims/hypothesis:**

We hypothesised that islet beta cell antigen presentation in the gut along with a tolerising cytokine would lead to antigen-specific tolerance in type 1 diabetes. We evaluated this in a parallel open-label Phase 1b study using oral AG019, food-grade *Lactococcus lactis* bacteria genetically modified to express human proinsulin and human IL-10, as a monotherapy and in a parallel, randomised, double-blind Phase 2a study using AG019 in combination with teplizumab.

**Methods:**

Adults (18–42 years) and adolescents (12–17 years) with type 1 diabetes diagnosed within 150 days were enrolled, with documented evidence of at least one autoantibody and a stimulated peak C-peptide level >0.2 nmol/l. Participants were allocated to interventions using interactive response technology. We treated 42 people aged 12–42 years with recent-onset type 1 diabetes, 24 with Phase 1b monotherapy (open-label) and 18 with Phase 2a combination therapy. In the Phase 2a study, after treatment of the first two open-label participants, all people involved were blinded to group assignment, except for the Data Safety Monitoring Board members and the unblinded statistician. The primary endpoint was safety and tolerability based on the incidence of treatment-emergent adverse events, collected up to 6 months post treatment initiation. The secondary endpoints were pharmacokinetics, based on AG019 detection in blood and faeces, and pharmacodynamic activity. Metabolic and immune endpoints included stimulated C-peptide levels during a mixed meal tolerance test, HbA_1c_ levels, insulin use, and antigen-specific CD4^+^ and CD8^+^ T cell responses using an activation-induced marker assay and pooled tetramers, respectively.

**Results:**

Data from 24 Phase 1b participants and 18 Phase 2a participants were analysed. No serious adverse events were reported and none of the participants discontinued AG019 due to treatment-emergent adverse events. No systemic exposure to AG019 bacteria, proinsulin or human IL-10 was demonstrated. In AG019 monotherapy-treated adults, metabolic variables were stabilised up to 6 months (C-peptide, insulin use) or 12 months (HbA_1c_) post treatment initiation. In participants treated with AG019/teplizumab combination therapy, all measured metabolic variables stabilised or improved up to 12 months and CD8^+^ T cells with a partially exhausted phenotype were significantly increased at 6 months. Circulating preproinsulin-specific CD4^+^ and CD8^+^ T cells were detected before and after treatment, with a reduction in the frequency of preproinsulin-specific CD8^+^ T cells after treatment with monotherapy or combination therapy.

**Conclusions/interpretation:**

Oral delivery of AG019 was well tolerated and safe as monotherapy and in combination with teplizumab. AG019 was not shown to interfere with the safety profile of teplizumab and may have additional biological effects, including changes in preproinsulin-specific T cells. These preliminary data support continuing studies with this agent alone and in combination with teplizumab or other systemic immunotherapies in type 1 diabetes.

**Trial registration:**

ClinicalTrials.gov NCT03751007, EudraCT 2017-002871-24

**Funding:**

This study was funded by Precigen ActoBio

**Graphical Abstract:**

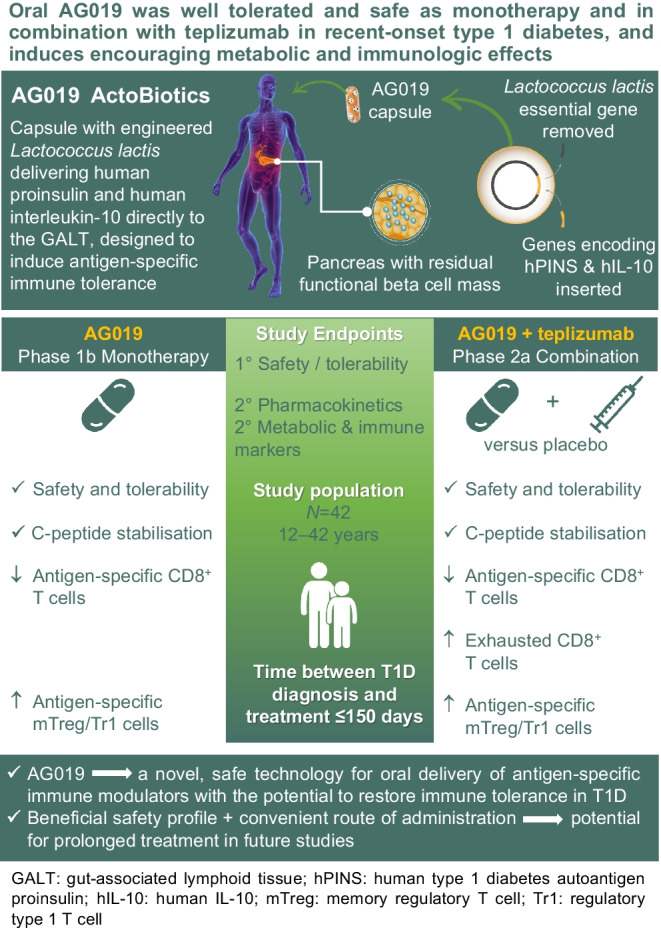

**Supplementary Information:**

The online version contains supplementary material available at 10.1007/s00125-023-06014-2.


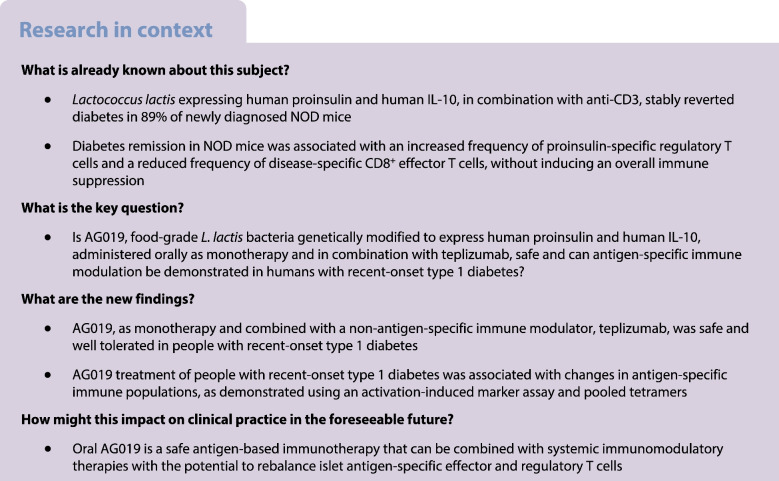


## Introduction

Type 1 diabetes is a T cell-mediated autoimmune disease with progressive destruction of insulin-producing beta cells in the pancreas [1]. Standard of care involves tight management of glycaemic control through life-long insulin therapy, but optimal metabolic management needed to prevent acute (e.g. ketoacidosis and severe hypoglycaemia) and chronic (e.g. retinopathy, nephropathy and heart disease) complications is not achieved in most people living with type 1 diabetes [2–5]. Immune-modulating disease-modifying therapies are progressively entering the field, with the first agent (the anti-CD3 monoclonal antibody teplizumab [Tzield]) approved by the Food and Drug Administration (FDA) for stage 2 diabetes (multiple autoantibody positivity and dysglycaemia). T cell-directed immune therapies, such as teplizumab, low-dose anti-thymocyte globulin and the fusion protein abatacept (cytotoxic T lymphocyte-associated antigen-4-immunoglobulin), and antigen-specific immune therapies, such as IMCY-0098, have shown promising results and are currently under clinical investigation for treatment of recent-onset type 1 diabetes [6]. Combining immune modulation with antigen introduction in a tolerogenic manner could lead to antigen-specific immune therapies, able to reset or arrest underlying disease processes, and is an appealing approach to treat type 1 diabetes with fewer off-target effects and a more sustainable efficacy compared with systemic immunosuppressive therapies [7].

Oral tolerance, using the oral route and the gut-associated lymphoid tissue to induce immunoregulatory mechanisms, has been successfully explored in other diseases such as arthritis [8]. The proposed mechanism of action of AG019, *Lactococcus lactis* bacteria delivering the human type 1 diabetes autoantigen proinsulin (hPINS) and human IL-10 (hIL-10) directly to the intestinal mucosa, is to re-educate immune cells towards antigen-specific tolerance without systemically impacting the natural protective functions of the peripheral immune system. Preclinical studies with AG019 in combination with a low dose of anti-CD3 monoclonal antibody stably reverted diabetes in up to 89% of newly diagnosed NOD mice [9–12]. Treatment was associated with increased frequencies and accumulation of insulin-reactive regulatory T cells (Tregs) in and around the pancreatic islets and a reduction in disease-specific autoreactive CD8^+^ T cells, without inducing generalised immune suppression.

Teplizumab, an Fc receptor non-binding humanised anti-CD3 antibody, demonstrated therapeutic efficacy in multiple clinical trials in recent-onset and stage 2 diabetes [13–19] by selectively inactivating and eventually eliminating pathogenic cells while preserving Tregs [20–23]. Importantly, teplizumab induces a state of exhaustion [13, 19, 20, 24–26], a mechanism of action that may be complementary to the antigen-specific AG019 immune therapy as indicated by the preclinical data in the NOD mice.

These preclinical and clinical data have been the basis of a first-in-human (FIH) Phase 1b/2a study with AG019, administered as monotherapy and in combination with teplizumab, in adults and adolescents with recent-onset type 1 diabetes and residual beta cell function. The main study objective was to assess the safety and tolerability of AG019. Secondary endpoints included pharmacokinetic (PK) data to examine AG019 exposure, metabolic data to examine functional beta cell preservation and glycaemic control, and immunological data to gain insight into the mode of action of AG019 in type 1 diabetes.

## Methods

We performed an FIH Phase 1b/2a study with AG019 and teplizumab in participants with recent-onset type 1 diabetes, recruited from 15 clinical sites in the USA and Belgium. The study protocol and its amendments, and the informed consent forms, were approved by an independent ethics committee and institutional review board. All participants/parents/guardians provided written informed consent before study entry. A data safety monitoring board reviewed the safety data at prespecified timepoints.

### Study population

Screening was done within 28 days of drug administration. Adults (aged 18–42 years) and adolescents (aged 12–17 years) with type 1 diabetes, according to ADA criteria [27], and diagnosed within 150 days, were enrolled. Enrolment required evidence of at least one autoantibody and a stimulated peak C-peptide level >0.2 nmol/l during a 4 h mixed meal tolerance test (MMTT). Ethnicity and sex were investigator assessed or self-reported.

### Study design

**Fig. 1:**
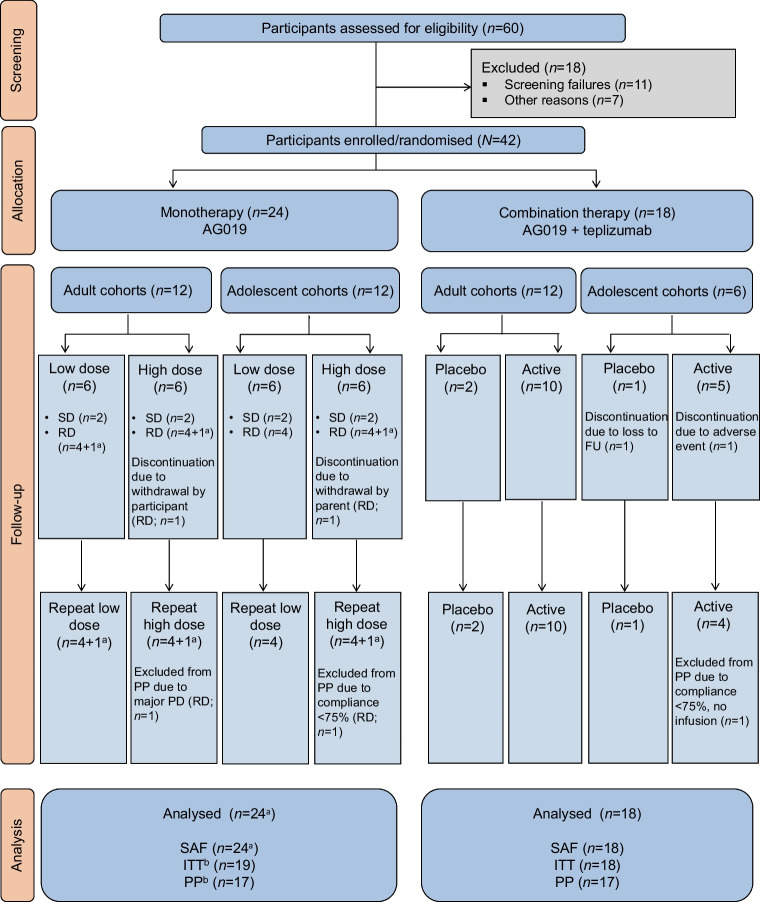
Participant disposition. ^a^A total of three single-dose participants were re-enrolled as repeat-dose participants in the different monotherapy cohorts. ^b^Single-dose participants were not included in the ITT and PP analysis sets. FU, follow-up; PD, protocol deviation; RD, repeat dose; SAF, safety analysis set; SD, single dose

The study consisted of two parts. In the open-label Phase 1b study, two single ascending doses (low and high dose: one or three capsules twice daily, respectively) of AG019 monotherapy were administered in adults and adolescents. Participants were enrolled in four sequential AG019 monotherapy cohorts (low-dose adult, high-dose adult, low-dose adolescent, high-dose adolescent) following a multi-step enrolment plan (electronic supplementary material [ESM] Fig. 1). Single-dose participants received AG019 on day 1 and were followed until the end-of-study visit on day 4. Repeat-dose participants started AG019 treatment on day 1 with repeated daily doses for 8 weeks and were followed for 12 months.

In the randomised, double-blind Phase 2a study, adult and adolescent participants were enrolled in the AG019/teplizumab combination therapy cohort. Three AG019 (or placebo) capsules were administered twice daily for 8 weeks, and teplizumab or placebo infusions were administered daily for the first 12 days. In each combination therapy cohort, the first two participants were enrolled in a staggered way as a safety measure and received active treatment (AG019 plus teplizumab) in an open-label fashion. The remaining ten participants were randomised (4:1) to active treatment or placebo in a double-blind fashion. Participants were randomised and started treatment on day 1 and were followed for 12 months (8 weeks of treatment and 10 months of post-treatment follow-up).

### Treatment

AG019 is an oral capsule formulation consisting of environmentally contained *L. lactis* bacteria (1×10^11^ colony forming units per capsule) genetically engineered to secrete hPINS and hIL-10. AG019 (or placebo) capsules were taken at home: two capsules (one in the morning, one in the evening) for low-dose participants in the Phase 1b study, and six capsules (three in the morning, three in the evening) for high-dose participants in the Phase 1b study and the participants in the Phase 2a study. Participants in the combination therapy cohorts received i.v. infusions of teplizumab (or placebo) at the study site for 12 days (total cumulative dose of ∼9034 µg/m^2^, supplied by Macrogenics, USA). All participants received diabetes management therapy with treatment-to-target goals according to ADA or equivalent guidelines [27].

### Safety assessments

The primary endpoint was the incidence of treatment-emergent adverse events (TEAEs), collected from all participants up to 6 months post treatment initiation. Safety data collected up to the 12 month follow-up visit were assessed as a secondary safety endpoint. Throughout the study, all repeat-dose participants recorded daily insulin use in an electronic diary.

### PK assessments

Systemic and local exposure to AG019 or its secreted proteins was assessed as a secondary endpoint. Blood and plasma samples were collected from all repeat-dose participants at screening, on day 12 and day 56 (last day of treatment), and at month 3 to evaluate the presence of AG019 in whole blood using plating and quantitative PCR (qPCR) and AG019-secreted hPINS and hIL-10 in plasma using ELISA. Faecal samples were collected from all repeat-dose participants in the high-dose monotherapy cohorts and all combination therapy cohorts at screening and on days 56, 58, 60, 62 and 64 to evaluate the presence of AG019 using qPCR.

### Pharmacodynamic assessments

Pharmacodynamic activity was assessed as a secondary endpoint by measurement of metabolic and immunological variables. Blood samples, collected from all repeat-dose participants in the monotherapy and combination therapy cohorts at screening, on day 56, and at months 3, 6, 9 and 12, were analysed for HbA_1c_ (%). C-peptide levels were measured during an MMTT performed at screening and at months 3, 6 and 12. These laboratory assessments were performed by Eurofins Central Laboratory (USA). Peripheral blood mononuclear cells were isolated from blood samples collected from all repeat-dose participants in the high-dose monotherapy and all combination therapy cohorts at screening; on days 1, 12 and 56; and at months 3, 6, 9 and 12 for assessment of antigen-specific (preproinsulin [PPI]-specific) CD4^+^ and CD8^+^ T cell populations. Antigen-specific CD4^+^ T cell populations were identified using an activation assay and analysed by flow cytometry on a CYTEK Aurora spectral cytometer (USA). Antigen-specific CD8^+^ T cells were stained using tetramers according to established methods [28] and analysed by cytometry by time of flight (CyTOF) on a Helios CyTOF mass cytometer (USA). A full list of peptides and antibodies is provided in ESM Tables 1 and 2, and the gating strategy is depicted in ESM Fig. 2.

### Statistical analysis

The safety analysis set comprised all 42 enrolled participants who received at least one AG019 dose. All repeat-dose participants in the AG019 monotherapy (*n*=19) and AG019/teplizumab combination therapy cohorts (*n*=18) were included in the intention-to-treat (ITT) analysis. The per protocol (PP) analysis set included all repeat-dose participants who received at least 75% of the scheduled AG019 doses and, in the combination therapy cohorts, at least one dose of teplizumab, and had no major protocol deviations. Two participants (one adult and one adolescent) from the high-dose monotherapy cohort and one participant who did not start teplizumab in the combination therapy cohort were excluded from the PP analysis. Enrolment of adolescents in the combination therapy cohort was prematurely terminated due to the impact of the COVID-19 pandemic.

Safety data were descriptively analysed based on the safety analysis set. ANOVA was used to compare demographic and baseline characteristics between all cohorts and between active and placebo groups in the AG019/teplizumab cohorts. PK data were descriptively analysed based on a subset of the ITT analysis set (no PK faecal samples were collected from low-dose monotherapy participants). All metabolic data analyses were based on the PP analysis set. The 2 h AUC of C-peptide from the MMTT was calculated using the linear trapezoidal rule over a 2 h period (0–120 min), and the C-peptide mean AUC was obtained by dividing the calculated AUC values by the time period (120 min). The 2 h mean C-peptide AUC was used to identify treatment responders post hoc according to published criteria [29, 30]; a participant was classified as a responder when the change from baseline (at 6 or 12 months) was either non-negative or, if negative, represented a CV ≤9.7%. HbA_1c_ and insulin use data were summarised using descriptive statistics and a post hoc analysis (paired *t* test vs baseline). CD4^+^ and CD8^+^ T cell analyses were based on a subset of the PP analysis set; no samples were collected from low-dose monotherapy participants. Changes from baseline in antigen-specific CD4^+^ T cell frequencies were analysed using the Wilcoxon matched-pairs signed-rank test. Mixed-effects analysis was used to compare antigen-specific CD8^+^ T cell frequencies over time. Unpaired *t* tests were used to compare frequencies (expressed as log fold change vs baseline) between treatment groups and a Spearman or Pearson correlation was used for assessment of bivariate data. All statistical tests were two-sided. Statistical significance was set at *p*<0.05. Immunological and metabolic analyses were performed using GraphPad Prism v9.0.2 (GraphPad Software, USA). Data are expressed as means ± SEM or as means ± SD.

Details of study population, sample size, interim analysis, randomisation and blinding, and pharmacodynamic assessments are provided in the ESM Methods.

## Results

### Participant disposition and baseline characteristics

The study was conducted between October 2018 and October 2021. Participant enrolment in Phase 1b (AG019 monotherapy) and Phase 2a (AG019/teplizumab combination therapy) is shown in Fig. 1. Sixty participants were screened, 18 of whom were excluded from participation in the study. Twenty-four participants were enrolled and treated in the AG019 monotherapy cohorts; two participants voluntarily withdrew after the 3 month visit. In the AG019/teplizumab combination therapy cohorts, 18 participants were enrolled and treated. All participants completed the study PP, except for one AG019/teplizumab adolescent (open-label) who did not start teplizumab infusions due to a non-TEAE and one placebo adolescent who was lost to follow-up after the 9 month visit.

All groups were equally balanced for age, sex, autoantibody positivity and ethnicity (Table 1), and the mean time from diagnosis to treatment start was 102.2 days. Participants’ baseline characteristics were similar in each study arm. All but one of the participants were taking insulin at study entry.

**Table 1:** Demographics and baseline characteristics

Characteristic	AG019 monotherapy				AG019/teplizumab combination therapy			
	Adults		Adolescents		Adults		Adolescents	
	RD low (*n*=5)	RD high (*n*=5)	RD low (*n*=4)	RD high (*n*=5)	Placebo (*n*=2)	Active (*n*=10)	Placebo (*n*=1)	Active (*n*=5)
Age (years)								
Mean	27.2	25.4	14.3	15.0	29.0	27.9	12.0	13.6
[SD]	[8.9]	[7.4]	[2.2]	[1.9]	[5.7]	[6.7]	–	[1.1]
Sex, *n* (%)								
Male	4 (80)	3 (60)	2 (50)	2 (40)	1 (50)	6 (60)	0 (0)	2 (40)
Female	1 (20)	2 (40)	2 (50)	3 (60)	1 (50)	4 (40)	1 (100)	3 (60)
Ethnicity, *n* (%)								
Hispanic or Latino	0 (0)	1 (20)	0 (0)	0 (0)	0 (0)	0 (0)	0 (0)	0 (0)
Not Hispanic or Latino	5 (100)	4 (80)	4 (100)	5 (100)	2 (100)	10 (100)	1 (100)	5 (100)
Time from diagnosis to treatment start (days)								
Mean	91.0	100.0	95.0	126.4	70.0	101.0	90.0	122.6
[SD]	[38.2]	[42.2]	[21.3]	[19.4]	[15.6]	[35.9]	–	[36.3]
HbA_1c_ (mmol/mol)								
Mean	46.2^a^	53.0	46.5	45.1	56.8	52.1	68.3	54.5
[SD]	[6.1]^a^	[17.1]	[5.6]	[9.7]	[33.2]	[15.5]	–	[26.6]
HbA_1c_ (%)								
Mean	6.38^a^	7.00	6.40	6.28	7.35	6.92	8.40	7.14
[SD]	[0.56]^a^	[1.57]	[0.51]	[0.89]	[3.04]	[1.42]	–	[2.43]
Insulin dose-adjusted HbA_1c_								
Mean	7.39^a^	8.25	9.09	8.39	8.53	8.41	10.64	9.61^a^
[SD]	[1.30]^a^	[2.37]	[1.09]	[2.47]	[4.54]	[1.73]	–	[2.58]^a^
C-peptide fasting (nmol/l)								
Mean	0.27	0.38	0.33	0.27^a^	0.36	0.18	0.16	0.25
[SD]	[0.15]	[0.20]	[0.08]	[0.14]^a^	[0.22]	[0.13]	–	[0.06]
C-peptide peak (nmol/l)								
Mean	0.92	1.26	0.97	0.81	1.10	0.71	0.28	0.76
[SD]	[0.35]	[1.00]	[0.45]	[0.21]	[0.11]	[0.26]	–	[0.29]
C-peptide mean 2 h AUC (nmol/l)								
Mean	0.62	0.89	0.78	0.57	0.73	0.48	0.25	0.57
[SD]	[0.28]	[0.61]	[0.35]	[0.13]	[0.01]	[0.19]	–	[0.21]
Insulin use (IU kg^–1^ day^–1^)								
Mean	0.23	0.39^a^	0.67	0.53	0.30	0.37	0.56	0.51^a^
[SD]	[0.18]	[0.20]^a^	[0.27]	[0.46]	[0.37]	[0.14]	–	[0.18]^a^
Insulin antibodies, *n* (%)^b^								
Negative	2 (40)	2 (40)	1 (25)	2 (40)	1 (50)	5 (50)	0 (0)	3 (60)
Positive	3 (60)	3 (60)	1 (25)	2 (40)	1 (50)	0 (0)	1 (100)	1 (20)
Missing	0 (0)	0 (0)	2 (50)	1 (20)	0 (0)	5 (50)	0 (0)	1 (20)
IA-2 antibodies, *n* (%)^b^								
Negative	2 (40)	1 (20)	2 (50)	2 (40)	1 (50)	5 (50)	0 (0)	1 (20)
Positive	2 (40)	4 (80)	2 (50)	2 (40)	1 (50)	3 (30)	1 (100)	4 (80)
Missing	1 (20)	0 (0)	0 (0)	1 (20)	0 (0)	2 (20)	0 (0)	0 (0)
GAD65 antibodies, *n* (%)^b^								
Negative	0 (0)	0 (0)	1 (25)	0 (0)	0 (0)	0 (0)	0 (0)	2 (40)
Positive	5 (100)	5 (100)	3 (75)	5 (100)	2 (100)	10 (100)	1 (100)	3 (60)
Missing	0 (0)	0 (0)	0 (0)	0 (0)	0 (0)	0 (0)	0 (0)	0 (0)
ZnT8 antibodies, *n* (%)^b^								
Negative	0 (0)	2 (40)	1 (25)	1 (20)	0 (0)	2 (20)	0 (0)	0 (0)
Positive	2 (40)	2 (40)	3 (75)	3 (60)	0 (0)	5 (50)	0 (0)	3 (60)
Missing	3 (60)	1 (20)	0 (0)	1 (20)	2 (100)	3 (30)	1 (100)	2 (40)
Cytomegalovirus antibodies, *n* (%)								
Negative	5 (100)	5 (100)	3 (75)	5 (100)	1 (50)	5 (50)	1 (100)	4 (80)
Positive	0 (0)	0 (0)	1 (25)	0 (0)	1 (50)	5 (50)	0 (0)	0 (0)
Missing	0 (0)	0 (0)	0 (0)	0 (0)	0 (0)	0 (0)	0 (0)	1 (20)
Epstein–Barr virus antibodies, *n* (%)								
Negative	2 (40)	4 (80)	4 (100)	4 (80)	0 (0)	4 (40)	1 (100)	4 (80)
Positive	3 (60)	1 (20)	0 (0)	1 (20)	2 (100)	6 (60)	0 (0)	1 (20)

All data are based on the safety analysis set in repeat-dose participants, except for C-peptide values, which are based on the ITT analysis set in repeat-dose participants

^a^*n*=4

^b^If evidence of autoantibody positivity to at least one beta cell autoantigen was documented in a participant’s medical file, the assessment was not required as part of eligibility verification

RD, repeat dose

### Safety and tolerability of AG019

No serious adverse events, deaths or TEAEs leading to discontinuation of AG019 treatment were reported.

TEAEs according to severity and to system organ class are summarised in ESM Tables 3 and 4, respectively. All TEAEs in Phase 1b were of Common Terminology Criteria for Adverse Events (CTCAE) grade 1 or 2; no severe TEAEs (grade ≥3) were reported. The majority of TEAEs (89.7%) were ‘not reasonably related to AG019’. There was no evidence of an effect of dose or age on incidence of adverse events. In Phase 2a, 95.3% of TEAEs were of CTCAE grade 1 or 2. Nine TEAEs of grade 3 or higher were reported in six AG019/teplizumab-treated participants (none in placebo). TEAEs reported as reasonably related to AG019 were mostly gastrointestinal (GI) disorders (diarrhoea and vomiting). One AG019/teplizumab-treated adult reported two grade 3 TEAEs considered reasonably related to AG019 and teplizumab (diarrhoea and vomiting) but AG019 or teplizumab treatment was not discontinued. In line with protocol-defined infusion-withholding criteria, teplizumab treatment was discontinued in five participants due to TEAEs (ESM Table 5); all participants continued AG019 treatment and completed the study. Transient changes in laboratory safety variables were observed in AG019/teplizumab-treated participants (including increases in liver function tests and decreases in lymphocyte, leucocyte and platelet counts), which were considered clinically significant and were reported as TEAEs in nine participants.

### AG019 PK analysis

AG019 bacteria were not detected in blood by plating or by qPCR, nor were there indications of AG019-related hPINS or hIL-10 in plasma measured by ELISA, either during treatment or 1 month after the last dose of AG019 (day 90).

**Fig. 2:**
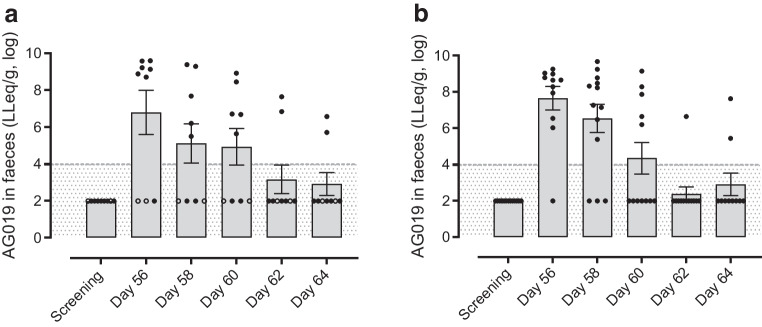
Concentrations of AG019 bacteria in faecal samples collected after the last day of AG019 dosing (day 56) from participants treated with (**a**) AG019 monotherapy or (**b**) AG019/teplizumab combination therapy. LLeq, *L. lactis* equivalents; grey area is below the limit of detection (LOD) (i.e. 9×10^3^ LLeq/g). Bacterial concentrations are expressed as LLeq/g and were log-transformed. Bars indicate means ± SEM. Faecal samples from 25 participants were analysed: nine AG019 monotherapy participants (five adults, four adolescents, all high-dose treated), 13 AG019/teplizumab combination therapy participants (ten adults, three adolescents) and three placebo participants (all negative; data not shown). Participants excluded from the PP analysis set are indicated in open symbols

Twenty-five participants (nine high-dose AG019-treated, 13 AG019/teplizumab-treated and three placebo-treated) provided faecal samples. AG019 bacteria were detected in faecal samples of 18/22 (82%) repeat-dose participants treated with AG019 high-dose monotherapy or AG019/teplizumab combination therapy at one or more post-screening sampling timepoints, indicating GI exposure to AG019 after oral dosing (Fig. 2). Of the four participants who lacked faecal bacterial recovery, two were excluded from the PP analysis set due to a major protocol deviation (the use of antibiotics, etc.) or AG019 compliance <75%. No AG019 bacteria were detected in the faecal samples from placebo-treated participants.

On day 64 (1 week after last AG019 dosing), AG019 was undetectable in 11/15 (73%) of the participants who provided a sample and had detectable levels of AG019 bacteria at one or more earlier timepoints. AG019 levels were detectable but strongly decreased (mean 2.9 log decrease from peak levels) in the other four participants.

### Effects of treatments on metabolic responses

**Fig. 3:**
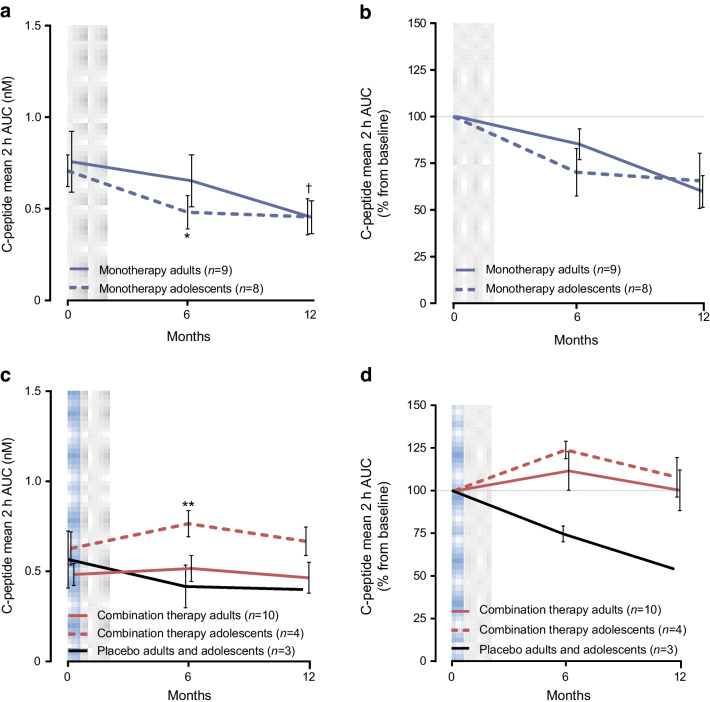
C-peptide mean 2 h AUC over time. (**a**) Absolute values in participants treated with AG019 monotherapy. (**b**) Percentage change from baseline in participants treated with AG019 monotherapy. (**c**) Absolute values in participants treated with AG019/teplizumab combination therapy and placebo. (**d**) Percentage change from baseline in participants treated with AG019/teplizumab combination therapy and placebo. Data are based on the PP analysis set and are means ± SEM. Pairwise comparison vs baseline (post hoc *t* test): *p<0.05 (in adolescents), **p<0.01 (in adolescents), ^†^*p*<0.05 (in adults). Grey shading indicates the AG019 treatment period; blue shading indicates the AG019+teplizumab treatment period

The C-peptide analysis results are shown in Fig. 3 and ESM Fig. 3. In the adult monotherapy cohort, the mean 2 h C-peptide AUC at 6 months was not significantly changed from baseline (85%) but declined at 12 months (60% of baseline, *p*=0.03 on absolute values). Among adolescents, the mean 2 h C-peptide AUC declined at 6 months (70% of baseline, *p*=0.044 on absolute values) and 12 months (66% of baseline, *p*=0.07 on absolute values). In the adult combination therapy cohort, the C-peptide response increased (112%) at 6 months and was unchanged (100%) at 12 months compared with declines in the placebo-treated group (73% and 54% of baseline, *n*=2). Similarly in adolescents, the C-peptide increased to 124% of baseline levels at 6 months (*p*=0.007) and 108% at 12 months vs 77% in the placebo-treated adolescent at 6 months (no data at 12 months).

**Fig. 4:**
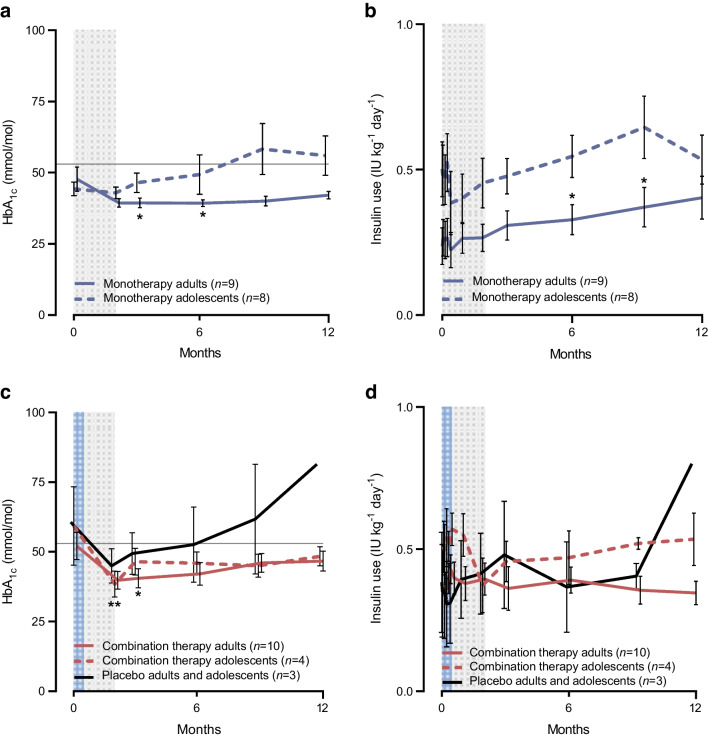
HbA_1c_ (**a**, **c**) and insulin use (**b**, **d**) over time in participants treated with (**a**, **b**) AG019 monotherapy or (**c**, **d**) AG019/teplizumab combination therapy and placebo. Data are based on the PP analysis set. Horizontal line for HbA_1c_ represents the target for glycaemic control (53 mmol/mol). Pairwise comparison vs baseline (post hoc *t* test): *p<0.05, **p<0.01 in adults. Data are means ± SEM. Grey shading indicates the AG019 treatment period; blue shading indicates the AG019+teplizumab treatment period

In the adult monotherapy cohort, the mean levels of HbA_1c_ declined during AG019 treatment and were significantly decreased as compared with baseline (*p*=0.036 and *p*=0.044 at 3 and 6 months, Fig. 4). In the adolescent monotherapy cohort, HbA_1c_ was not significantly changed from baseline. Daily insulin use increased from baseline by 0.17 IU kg^−1^ day^−1^ in AG019 monotherapy-treated adults and by 0.02 IU kg^−1^ day^−1^ in adolescents at 12 months. In AG019/teplizumab-treated adults and adolescents, there was a decrease in HbA_1c_ from baseline to month 12. In adults receiving the combination therapy, the levels were significantly lower at months 2 (*p*=0.009) and 3 (*p*=0.038). At 12 months, the mean daily insulin use decreased as compared with baseline (by −0.03 IU kg^−1^ day^−1^) in AG019/teplizumab-treated adults and increased by 0.03 U kg^−1^ day^−1^ in adolescents.

### Effects of treatment on total and antigen-specific CD4^+^ and CD8^+^ T cells

**Fig. 5:**
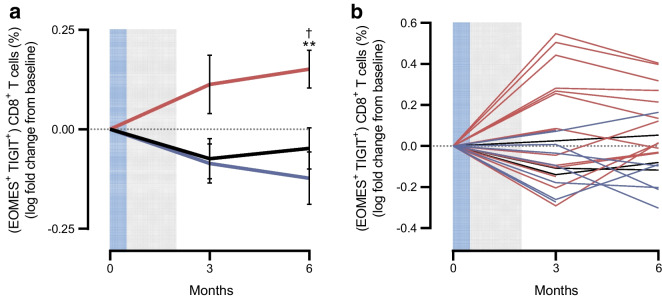
Frequency of partially exhausted (EOMES^+^TIGIT^+^) CD8^+^ T cells over time in participants treated with AG019 monotherapy, AG019/teplizumab combination therapy and placebo. (**a**) Data per treatment group. (**b**) Individual data. Data are based on a subset of the PP analysis set; adults and adolescents are included together. Percentages were normalised to baseline using the log fold change and data are means ± SEM. Mixed effect analysis vs baseline: ^†^*p*<0.05; unpaired *t* test vs AG019: **p<0.01. Blue lines, monotherapy; red lines, combination therapy; black lines, placebo. Grey shading indicates the AG019 treatment period; blue shading indicates the AG019+teplizumab treatment period

We previously reported that teplizumab induced CD8^+^ T cells that express the *KLRG1*, *TIGIT* and *EOMES* genes, and found that higher levels after treatment were associated with clinical responses to the drug [13, 26]. Consistent with past results, these CD8^+^ T cells were increased in the AG019/teplizumab combination therapy group compared with baseline at month 6 (*p*=0.013), but not in the participants treated with AG019 alone or placebo (Fig. 5).

**Fig. 6:**
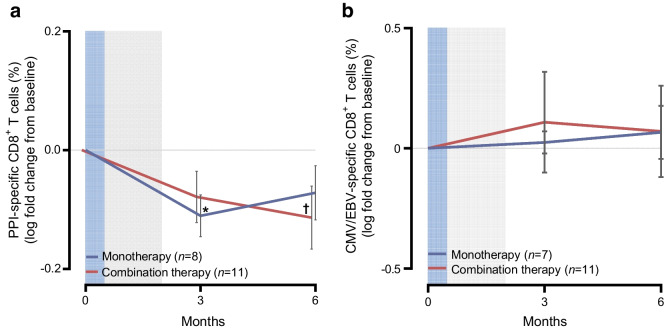
Frequency of (**a**) PPI-specific CD8^+^ T cells and (**b**) CMV/EBV-specific CD8^+^ T cells over time in participants treated with AG019 monotherapy and AG019/teplizumab combination therapy. Data are based on the PP analysis set. Percentages were normalised to baseline using the log fold change and data are means ± SEM. Mixed effect analysis vs baseline: *p<0.05 (AG019 monotherapy), ^†^*p*<0.05 (AG019/teplizumab combination therapy). Grey shading indicates the AG019 treatment period; blue shading indicates the AG019+teplizumab treatment period. CMV/EBV, cytomegalovirus/Epstein–Barr virus

Based on preclinical data in NOD mice, we analysed the frequencies of antigen-specific CD8^+^ T cells and two populations of regulatory CD4^+^ antigen-specific T cells. The frequency of PPI-specific CD8^+^ T cells significantly decreased from baseline by 22.5% (*p*=0.016) at 3 months in AG019 monotherapy participants and by 21.6% (*p*=0.035) at 6 months in the combination group (Fig. 6). In the placebo group there was an average 12.5% increase at 3 months and a 17.1% reduction at 6 months. There was no significant change in the frequency of cytomegalovirus/Epstein–Barr virus viral-specific CD8^+^ T cells in either treatment group (Fig. 6), and no correlation was found between the change in PPI-specific CD8^+^ T cell frequency and age.

**Fig. 7:**
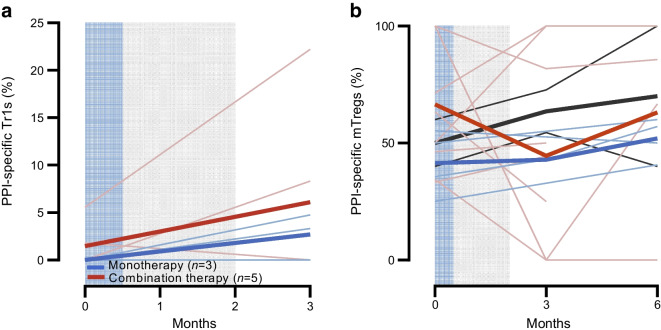
Frequency of PPI-specific (**a**) Tr1s and (**b**) memory Tregs (mTregs) in adults treated with AG019 monotherapy or AG019/teplizumab combination therapy. (**a**) The frequency of PPI-specific IL-10^+^ Tr1s in adults treated with AG019 monotherapy (*n*=3) or AG019/teplizumab combination therapy (*n*=5) at baseline and at month 3. Thick lines represent the mean frequency and thin lines depict the frequency of cells for each participant. (**b**) The frequency of PPI-specific mTregs in adults treated with monotherapy (*n*=4) or combination therapy (*n*=7) at baseline, 3 months and 6 months as described in (**a**). Two adult participants treated with placebo are depicted in black. The parent population for Tr1s and mTregs is total PPI-specific CD4^+^ T cells. Grey shading indicates the AG019 treatment period; blue shading indicates the AG019+teplizumab treatment period

There was a modest increase in the frequency of PPI-specific IL-10^+^ regulatory type 1 T cells (Tr1s) in adults treated with monotherapy (0% at baseline, 2.7% at 3 months) and with combination therapy (2.3% at baseline, 4.1% at 3 months, Fig. 7a), but not in placebo-treated participants. Responses to control (viral and bacterial) antigens were unchanged. The PPI-reactive Tr1s were below the limit of detection in adolescent participants. The frequency of PPI-specific CD4^+^ memory Tregs showed a similar increase in adult participants treated with monotherapy and with combination therapy (Fig. 7b), whereas this was not seen in adolescents.

### Association of T cell changes with C-peptide preservation

**Fig. 8:**
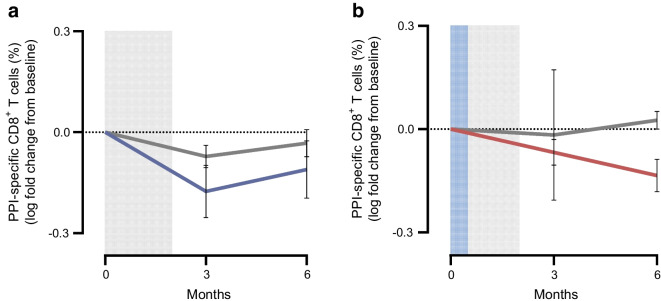
PPI-specific CD8^+^ T cells over time in clinical responders and non-responders at 6 months in (**a**) AG019 monotherapy and (**b**) AG019/teplizumab combination therapy. Data are based on a subset of the PP analysis set and are presented as means ± SEM. AG019 monotherapy responders (*n*=3, blue); AG019 monotherapy non-responders (*n*=4, grey); AG019/teplizumab combination therapy responders (*n*=11, red); AG019/teplizumab combination therapy non-responders (*n*=2, grey). Grey shading indicates the AG019 treatment period; blue shading indicates the AG019+teplizumab treatment period

We designated post hoc clinical responders as participants who lost ≤9.7% of their baseline C-peptide at month 6 based on published criteria and examined a correlation with immunological results. Seven of 16 (44%) participants (5/9 adults and 2/7 adolescents) in the AG019 monotherapy group were classified as responders at 6 months. In AG019/teplizumab combination therapy, there were 11/14 (79%) responders (7/10 adults and 4/4 adolescents) at month 6 and 0/3 in the placebo group (ESM Table 6, ESM Fig. 4). In AG019 monotherapy, the frequency of PPI-specific CD8^+^ T cells was lower in the responders vs non-responders at 3 months (mean decrease from baseline of 33% in responders vs 15% in non-responders). In AG019/teplizumab combination therapy, antigen-specific T cells showed a 27% reduction in the frequency of PPI-specific CD8^+^ T cells in the responders compared with an increase of 6% in the non-responders at 6 months (Fig. 8).

## Discussion

Combination therapies of immune modulation and antigen are appealing in organ-specific autoimmune diseases like type 1 diabetes. We demonstrated the safety and biological activity of an 8 week treatment of oral AG019 (*L. lactis* producing hPINS and hIL-10) in adults and adolescents with recent-onset type 1 diabetes. Also, we established the safety and biological activity when used as a combination treatment with teplizumab, a compound now approved for delay of type 1 diabetes in children and adults at risk of diabetes [13]. Dosing of AG019 was safe and effective since the bacteria were confirmed in the faeces of the repeat-dose participants in the high-dose monotherapy and combination therapy cohorts, without evidence for systemic exposure and with no TEAEs leading to AG019 treatment discontinuation. This favourable safety profile of AG019 provides an opportunity for a chronic or long-term treatment duration which, our data suggest, may be needed to sustain the effects of AG019. Because of its safety, AG019 could also be used at early stages in the development of type 1 diabetes, when the progression of disease is intermittent and slow. While this may sound like an attractive option for clinicians, specific measures to promote compliance, in particular in young individuals, will need to be put in place considering the duration of the therapy.

The rationale for the development of AG019 and for this clinical investigation was that hIL-10 locally delivered at the intestinal mucosa could induce IL-10-producing CD4^+^ Tr1s [31–34], thereby creating a local environment favouring tolerance. The co-delivery of hPINS by AG019 could facilitate the expansion of the Treg repertoire towards the major pancreatic autoantigen. The primary study objective was to assess safety and tolerability; therefore, the study was not powered for metabolic or immunologic efficacy. Nonetheless, there was an encouraging effect on metabolic markers for beta cell function and glycaemic control (2 h C-peptide AUC after MMTT, HbA_1c_ and insulin use), particularly in adults treated with AG019 monotherapy up to 6 months and in adults and adolescents treated with AG019/teplizumab combination therapy up to 12 months. The changes in C-peptide in the placebo group as compared with combination therapy indicate these increases or lack of decline are suggestive of a true effect of combination therapy and are not due to the ‘honeymoon phase’, a phase after type 1 diabetes diagnosis in which the pancreas is still able to produce insulin. Our findings include a non-significant increase in PPI-specific (IL-10-producing) Tr1s and Tregs in those treated with monotherapy and combination therapy. Concomitantly, there was a significant reduction in PPI-specific, but not viral-specific, CD8^+^ T cells at 3 months in AG019 monotherapy and at 6 months in AG019/teplizumab combination therapy, indicating the preservation of viral responses and suggesting the presence of antigen-specific immune modulation. These changes in immune markers were found in the peripheral blood and, because of the intestinal delivery of AG019, the effects on immune cells within the GI tract and potentially pancreas or draining lymph nodes may be greater [11, 35]. Altogether, the immunological data suggest that AG019 induces antigen-specific Tregs, which mimics what we previously demonstrated in newly diagnosed NOD mice [9–12]. We speculate that the combination with teplizumab (temporarily) pauses the autoimmune destruction of the remaining functional beta cell mass, allowing AG019 to install an islet-specific Treg repertoire which is known to be more suitable for controlling autoimmunity than polyclonal Tregs [35].

The complementary mode of action of teplizumab to the AG019-induced antigen-specific tolerogenic milieu can occur on multiple levels. Anti-CD3 increases Tr1s locally in the GI tract [36] and generates a ‘gut-homing’ CD4^+^CD25^hi^CCR6^+^FoxP3^+^ Treg population [23], thereby promoting the interaction between Tr1s/Tregs and the AG019-delivered IL-10/hPINS at the intestinal mucosa and creating the opportunity for antigen-specific immune modulation. Also, anti-CD3 therapy promotes tolerance by selectively eliminating pathogenic T cells while preserving Tregs [20], resulting in an enrichment of the regulatory compartment. The most robust and clinically relevant reported effect of teplizumab seems to be the expansion of a (partially) exhausted phenotype among total CD8^+^ T cells, an effect also observed in the AG019/teplizumab combination treatment group of the current study. This exhaustion profile, characterised by the expression of inhibitory receptors (killer cell lectin-like receptor G1 [KLRG1], T cell immunoreceptor with Ig and ITIM domains [TIGIT] and eomesodermin [EOMES]), limited cytokine production and reduced proliferative capacity, results in an effector T cell population with an altered functional response, and is correlated with a better metabolic outcome [13, 26] and slower disease progression [28, 37].

In addition to the biological activity of AG019 monotherapy, this is the first study examining combination therapy with teplizumab in humans. Combination therapies, either simultaneously or sequentially combining immune modulation with beta cell protective interventions, have been proposed as strategies to help sustain the stabilising effect of teplizumab on functional beta cell mass [38]. In NOD mice, combining T cell-targeting immune modulation with antigen-based interventions has been shown to provide long-term protection, persisting after stopping all therapy. Here we demonstrate in humans not only that this combination, with *L. lactis* secreting proinsulin and hIL-10, is safe, but also that the first indications of metabolic and immune effects were present. Importantly, we showed that the effects of AG019 are specific for the relevant antigen, and, therefore, the safety for chronic use of this biologic is supported. In this regard, AG019 could also be combined with other immune modulators, acting as inducers, creating a therapeutic window for the installation of the antigen-specific immune response [39]. Because of its safety, AG019 could even be used at early stages in the development of type 1 diabetes when the progression of disease is intermittent and slow.

This study has a few limitations that restrict conclusions. First, sample sizes are small in all of the groups, and we did not have a teplizumab-only group to compare the findings with for antigen-specific T cells or metabolic effects. For immune measures, while assay technical variation has been validated for islet antigen-specific CD8^+^ T cells (CV of ~20%; 28), natural biological variation in islet-specific T cell populations is not well established in the literature, and the placebo group was too small (*n*=3) for meaningful comparisons. Samples were limited, so functional assays were not performed to confirm changes in function of antigen-specific cells. Potential depletion early in the monotherapy group for PPI-specific CD8^+^ T cells, as determined by a reduction in absolute numbers, was suggested but needs to be confirmed in future studies along with additional functional studies. We also had a limited duration of follow-up, and it would be important to follow the antigen-specific Tr1s and Tregs to determine whether they decline or persist when the treatment is withdrawn.

In summary, AG019 provides a new technology for oral delivery of antigen-specific immune modulators that is safe and induces biological activity. The beneficial safety profile and the convenient route of administration open paths for prolonged AG019 treatment in future studies, which will further address the potential beneficial therapeutic effects for the treatment of type 1 diabetes.

### Supplementary Information

Below is the link to the electronic supplementary material.

Supplementary file1 (PDF 1.14 MB)

## Data Availability

The data that support the findings of this study are not openly available for reasons of sensitivity. The sponsor may, at its discretion, grant access to the data (or portions thereof) to qualified researchers on reasonable request; however, access may be subject to certain use and/or privacy obligations.

## Abbreviations

CTCAE: Common Terminology Criteria for Adverse Events
CyTOF: Cytometry by time of flight
EOMES: Eomesodermin
FIH: First-in-human
GI: Gastrointestinal
hIL-10: Human IL-10
hPINS: Human type 1 diabetes autoantigen proinsulin
ITT: Intention-to-treat
KLRG1: Killer cell lectin-like receptor G1
MMTT: Mixed meal tolerance test
PK: Pharmacokinetic
PP: Per protocol
PPI: Preproinsulin
qPCR: Quantitative PCR
TEAE: Treatment-emergent adverse event
TIGIT: T cell immunoreceptor with Ig and ITIM domains
Tr1: Regulatory type 1 T cell
Treg: Regulatory T cell
