# Supplementary material for: A first-in-human, open-label Phase 1b and a randomised, double-blind Phase 2a clinical trial in recent-onset type 1 diabetes with AG019 as monotherapy and in combination with teplizumab
Source: Diabetologia. 2023 Oct 2;67(1):27–41. doi: 10.1007/s00125-023-06014-2 (PMC10709251; doi:10.1007/s00125-023-06014-2)
Supplement: Supplementary file 1 — Supplementary file1 (PDF 1.14 MB) [file 125_2023_6014_MOESM1_ESM.pdf]

ESM Methods

Study population

Ethnicity of participants was determined by means of a digital questionnaire, to examine the effect of AG019 between Hispanic or non-Hispanic people with type 1 diabetes.

Sample size

The primary study objective of this study was to assess the safety and tolerability of AG019. The sample size in this FIH study was therefore not based on statistical considerations, but at least 4 participants per dose level in the AG019 monotherapy cohorts and 12 participants per dose level in the combination therapy cohorts were considered sufficient to evaluate safety and biological activity of AG019. A maximum of 48 patients were enrolled in clinical sites in the Unites States of America and Belgium.

Interim analyses

Following interim analyses were performed:

- After the last Day 180 follow-up visit of the last participant enrolled into the Phase 1b part of the study, a preliminary analysis of the data collected up to 6 months was performed for these participants.
- Once all participants enrolled in the adult combination therapy cohort, as well as the 2 participants enrolled into the open-label part of the adolescent combination therapy cohort, had completed the Day 180 follow-up visit, unblinding was done and an interim analysis was performed.
- After the last Day 180 follow-up visit of the last patient participant into the adolescent combination therapy cohort, all participants in this cohort were unblinded and an interim

analysis was performed on all, which included an analysis of the primary endpoint of the study.

All decisions on data for blinded participants were taken before breaking the blind for the third interim analysis. Since this is a study of descriptive nature, with no statistical tests, the influence of the interim analyses was considered negligible.

Randomization and blinding

All participants were registered in and randomization was performed through the Interactive Response Technology. Blinding of participants and all study personnel at the site was achieved using identical packaging for both active and placebo treatments.

Lab assessments

The lab assessments have been performed by Eurofins BioPharma Services using validated methods. Glucose was measured using a Roche Glucose kit based on hexokinase enzymatic reaction (spectrophotometry); intra CV ranged from 0.8-1.7% and inter CV ranged from 2.9-4.7%. HbA1c was measured on a BioRad Variant II Classic system using a dedicated BioRad HbA1c kit. The methodology was based on HPLC Ion Exchange (chromatography) and CV ranged from 0.4-3.1% (intra-CV) and from 1.4-4.8% (inter-CV). C-Peptide was measured on Siemens Centaur XP system using dedicated Siemens C-Peptide kit. The method was based on immunologic reaction followed by chemiluminescence detection. The intra CV ranged from 3.4-3.9% and inter CV was 5.7%.

Analysis of antigen specific CD4$^+$ T cells in the circulation

PBMCs were plated (5 x 10^6 cells/well) in the presence of anti-CD40 blocking antibody and MHC Class II optimized peptides from preproinsulin (PPI; for a full list of peptides, see **ESM Table 1**).

As a positive control for the assay, PBMCs were stimulated with a pool of 176 peptides from viral and bacterial antigens relevant to humans (CEFX, IDT Peptides). DMSO was used as a negative control. Cells were stimulated with peptides or DMSO for 18h at 37°C to allow for CD154 and CD137 upregulation on activated cells. The following day, stimulated cells were harvested, stained with an CD154-PE antibody and anti-PE coupled-magnetic beads were added. $CD154^{+}$ cells were enriched over a magnetic column, surfaced stained with antibodies for identification of $CD4^{+}$ conventional T cells (Tconv) and type 1 (inducible) regulatory T cells (Tr1 cells), and were fixed and permeabilized for intracellular cytokine staining. The column flow-through (containing CD154- cells) was then stained with a CD137-PE-Cy7 antibody, anti-PE magnetic beads were added, and cells were enriched over a second magnetic column. The eluted cells were surface stained with antibodies to identify $CD4^{+}$ Tregs and were fixed and permeabilized for cytokine and transcription factor staining (for a full list of antibodies, see **ESM Table 2)**. Cells were analysed on a CYTEK Aurora spectral cytometer, data unmixed, and analysed using FlowJo v10.7.1.

Antigen responsive cells were gated as $CD4^{+}CD154^{+}CD69^{+}$ (Tconv, Tr1) or $CD4^{+}CD154^{-}CD137^{+}CD25^{+}$ (Treg) using the DMSO negative control for each sample to set the gate for antigen responsive cells. Tr1 cells were gated based on co-expression of $CD226^{+}LAG3^{+}CD49b^{+}IL\text{-}10^{+}$ and Treg cells were gated as $CD25^{hi}CD127^{low}FOXP3^{+}Helios^{+}$. Memory antigen-specific Treg and Tconv cells were gated as $CD45RA^{-}CD45RO^{+}$. To calculate the absolute number of antigen-reactive cells per million CD4 T cells, an aliquot (1/50) of cells was taken before the harvested cells were enriched for CD154 and CD137 expression. The frequency was calculated as: (#Ag stimulated enriched cells x 10^{6})/(#CD4 T cells in pre-sample x dilution factor).

Microbial antigen and DMSO control data were determined in parallel to the PPI-specific $CD4^+$ T-cell analysis. The microbial antigen control was used as a positive control for the assay and as a non-islet T cell response in relation to PPI T cell frequency and phenotype. The DMSO negative control was used to set the flow cytometry gate for T cells responding to peptides, *i.e.,* responses above the DMSO background.

Analysis of antigen specific $CD8^+$ T cells in the circulation

Thawed cryopreserved PBMC samples (2 x 10^6 cells) from each time point for a single subject were individually stained with unique metal isotope labelled CD45 antibodies. All time points for a single subject were then combined, washed twice with PBS and stained for viability using cisplatin, followed by quenching and washing with protein-containing media. Cells were then pretreated with dasatinib and washed prior to staining with 50 µL solution containing 1 µL of each Class I Tetramer (Tmr) pool in running buffer for 15 minutes at 37°C. Without washing, 50 µL of a frozen surface antibody cocktail was added, and the sample was incubated for an additional 30 minutes at 4°C. Samples were washed, fixed, and stained with a frozen intracellular antibody cocktail. Subsequently, samples were washed, fixed, and stored at 4°C overnight or for up to 1 week prior to acquisition. On the day of acquisition, cells were washed and resuspended in cold ultrapure water containing 1/5th EQ Four Element Calibration Beads by volume and acquired on a Helios CyTOF mass cytometer with a target cell acquisition of 1,000,000 live events at a rate of 500 events/second. Files were converted to .FCS and then randomized and normalized for EQ bead intensity using the CyTOF Software. FlowJo software v10.7.1 was used to export .FCS files and to gate $CD8^+$ T cell and Tmr^+ populations.

In a separate analysis, $CD8^+$ T cells (both total and PPI-specific) were manually gated in FlowJo for EOMES and TIGIT staining, identifying a population of partially exhausted $EOMES^+TIGIT^+$ $CD8^+$ T-cells.

Changes in the frequency of (antigen-specific) $CD8^+$ T cells are reported as the log of the ratio of the frequency at a time point (month 3 or 6) to the frequency at month 0 (fold-change, FC). For this analysis, adult and adolescent patients were grouped and all available patients from the PD-PP population were included. One patient (an adult from the combination therapy group) had no HLA compatible with the PPI Tmr, and the frequency of Tmr^+ cells was below LOQ at all time points. Thus, this patient was not reported. Two patients from the combination therapy group were assayed at 2 months; these data were incorporated into the 3-month time point results.

ESM Table 1. Peptides used for antigen specific T cell detection.

	Protein	Sequence	Position	Length	HLA
Activation (AIM)	Preproinsulin	MALWMRLLPLLALLALWGPD	1 - 20	20	NA
		PLLALLALWGPDPAAAFVNQ	9 - 28	20	NA
		WGPDPAAAFVNQHLCGSHLV	17 - 36	20	NA
		FVNQHLCGSHLVEALYLVCG	25 - 44	20	NA
		SHLVEALYLVCGERGFFYTP	33 - 52	20	NA
		LVCGERGFFYTPKTRREAED	41 - 60	20	NA
		FYTPKTRREAEDLQVGQVEL	49 - 68	20	NA
		EAEDLQVGQVELGGGPGAGS	57 - 76	20	NA
		QVELGGGPGAGSLQPLALEG	65 - 84	20	NA
		GAGSLQPLALEGSLQKRGIV	73 - 92	20	NA
		ALEGSLQKRGIVEQCCTSIC	81 - 100	20	NA
		RGIVEQCCTSICSLYQLENY	89 - 108	20	NA
		TSICSLYQLENYCN	97 - 116	14	NA
Class I Tmr	Preproinsulin	ALWGPDPAAP	15-24	10	A2
		PLALEGSLQK	79-88	9	A3
		WLMRLLPLLAL	4-13	10	B7
		LPLLALLAL	8-16	8	B7
		ALWMLLLPL	2-10	9	B8
	CMV/EBV	CMV pp50: VTEHDYLLY	245-253	9	A1
		CMV pp65: NLUPMUATV	495-503	9	A2
		CMV pp65: QYDPVAALF	341-349	9	A24
		CMV pp65: TPRVTGGGAM	417-426	10	B7
		CMV IE1: ELRRKMMYK	199-207	9	B8
		EBV LMP2: CLGGLLTMV	426-434	10	A2
		EBV BMLF1: DYNPVKQLF	320-328	9	A24
		EBV EBNA3A: FLRGAYGL	325-333	8	B8

NA: Not applicable, CMV: cytomegalovirus, EBV: Epstein-Barr virus, Tmr: tetramer, AIM: Activation-Induced Marker

Tmr specificities are labelled with two metals, but not a third Tmr metal and pooled for staining and analyses.

ESM Table 2. Antibodies used for cytokine analysis.

Panel	Fluorophore/Metal	Specificity	Clone	Supplier
**Spectral CD4-Tr1**	BB515	CCR7	3D12	BD Biosciences
	viobrightFITC	CXCR3	REA232	Miltenyi
	BB700	IL-2	MQ1-17H12	BD Biosciences
	PerCP-ef710	IL-10	JES3-9D7	eBioscience
	PE	CD154	5C8	Miltenyi
	PE-CF594	LAG3	11C3C65	Biolegend
	PE-Cy7	CD137	4-1BB	Biolegend
	APC	CD69	FN50	Biolegend
	AF647	CTLA4	BNI3	Biolegend
	AF700	CD45RA	HI100	Biolegend
	APC-Cy7	PD-1	EH12.2H7	Biolegend
	BV421	CCR4	L291H4	Biolegend
	ef450	TNFa	MAb11	eBioscience
	BV480	TIGIT	741182	BD Biosciences
	BV510	CCR6	11A9	BD Biosciences
	BV570	CD45RO	UCHL1	Biolegend
	BV605	CD4	RPA-T4	Biolegend
	BV650	CD27	O323	Biolegend
	BV711	CD25	2A3	BD Biosciences
	BV750	CD49b	AK7	BD Biosciences
	BV785	CD95	DX2	Biolegend
	BUV395	CD38	HB7	BD Biosciences
	BUV450	LIVE/DEAD BLUE		Thermo fisher
	BUV496	CD3	UCHT1	BD Biosciences
	BUV563	CD226	DX11	BD Biosciences
	BUV661	HLA-DR	G46-6	BD Biosciences
	BUV737	CD14	M5E2	BD Biosciences
	BUV737	CD19	SJ25C1	BD Biosciences
	BUV737	CD56	NCAM16.2	BD Biosciences
**Spectral CD4 Treg**	AF488	Helios	22F6	Biolegend
	PerCP-ef710	IL-10	WD1928	ebioscience
	PE	CD154	5C8	Miltenyi
	PE-Dz594	LAG3	11C3C65	Biolegend
	PE/Cy5	Foxp3	236A/E6	ebioscience
	PE-Cy7	CD137	4-1BB	Biolegend
	APC	CD69	FN50	Biolegend
	AF647	KLRG1	SA231A2	Biolegend
	AF700	CD45RA	HI100	Biolegend
	APC-Cy7	CCR7	G043H7	Biolegend
	BV421	TIM3	F38-2E2	Biolegend
	ef450	CD127	eBioRDR5	eBioscience
	BV480	TIGIT	741182	BD Biosciences
	BV510	PD-1	EH12.1	BD Biosciences
	BV570	CD45RO	UCHL1	Biolegend

	BV605	CD4	RPA-T4	Biolegend
	BV750	CD27	O323	Biolegend
	superbright702	GARP	G14D9	eBioscience
	BV785	CTLA4	BNI3	Biolegend
	BUV395	CD38	HB7	BD Horizon
	BUV450	LIVE/DEAD BLUE		Thermo fisher
	BUV496	CD3	UCHT1	BD Horizon
	BUV563	CD25	2A3	BD Horizon
	BUV661	CD39	TU66	BD Opti
	BUV737	CD14	M5E2	BD Horizon
	BUV737	CD19	SJ25C1	BD Horizon
	BUV737	CD56	NCAM16.2	BD Horizon
	BUV805	CD8	SK1	BD Horizon
**CyTOF Tmr**	89Y	CD45	HI30	Standard BioTools
	106Cd	CD8a	RPA-T8	Biolegend*
	110Cd	CD3	UCHT1	Biolegend*
	111Cd	CD45	HI30	Standard BioTools
	112Cd	CD45	HI30	Standard BioTools
	113Cd	CD4	RPA-T4	Biolegend*
	114Cd	CD11c	Bu15	Biolegend*
	116Cd	HLA-DR	L243	Biolegend*
	141Pr	Granzyme B	QA16A02	Biolegend*
	142Nd	CD57	HCD57	Standard BioTools
	143Nd	CD45RA	HI100	Standard BioTools
	144Nd	CD38	HIT2	Standard BioTools
	145Nd	α4β7	NA	ENTYVIO
	146Nd	KIR2DL1	HP-MA4	Biolegend*
	146Nd	KIR2DL2	DX27	Biolegend*
	146Nd	KIR3DL1	DX9	Biolegend*
	147Sm	KLRG1	14C2A07	Biolegend*
	148Nd	CD14	M5E2	Biolegend*
	149Sm	CD127	A019D5	Standard BioTools
	150Nd	CX3CR1	2A9-1	eBioscience*
	151Eu	Helios	22F6	Biolegend*
	152Sm	CD2	TS1/8	Biolegend*
	153Eu	Lag3	11C3C65	Biolegend*
	154Sm	TIGIT	MBSA43	Standard BioTools
	155Dy	Tcf1/7	7F11A10	Biolegend*
	156Gd	CD25	M-A251	Biolegend*
	158Gd	CD27	L128	Standard BioTools
	159Tb	CD161	HP3G10	Standard BioTools
	160Gd	T-bet	4B10	Standard BioTools
	161Dy	CD39	A1	Biolegend*
	162Dy	Eomes	WO1928	eBioscience*
	163Dy	CXCR3	G025H7	Standard BioTools
	164Dy	CD95	DX2	Standard BioTools
	165Ho	CD19	HIB19	Standard BioTools
	166Er	CD49b	P1E6-C5	Biolegend*

	167Er	CCR7	G043H7	Standard Bio Tools
	168Er	Tmr		
	169Tm	Tmr		
	170Er	CD122	TU27	Standard BioTools
	171Yb	CD103	Ber-ACT8	Biolegend*
	172Yb	Ki-67	B56(Ki-67)	Biolegend*
	173Yb	TCRgd	B1	Biolegend*
	174Yb	Tmr		
	175Yb	PD1	EH12.2H7	Standard BioTools
	176Yb	CD56	NCAM16.2	BD Biosciences*
	191Ir	Iridium intercalator		Standard BioTools
	193Ir	Iridium intercalator		Standard BioTools
	198Pt	Cisplatin		Standard BioTools
	209Bi	CD16	3G8	Standard BioTools

For CyTOF: Unlabeled purified antibodies were conjugated to metal isotopes using Maxpar X8 and MCP9 Antibody Labeling Kits (Standard BioTools) as per manufacturer's instructions. All samples from the same subject were barcoded and combined for staining. Tmr gating was applied to all samples in the same subject, guided by subjects that were negative for TMr HLA's (Tmr+<0.03%, LOQ). Double labelled cells were counted, single and triple labelled cells were excluded.

ESM Table 3. Summary of treatment emergent adverse events according to severity

A: AG019 monotherapy cohorts (Phase 1b)

	**Adolescents, low dose n=4**		**Adolescents, high dose n=5**		**Adults, low dose n=5**		**Adults, high dose n=5**	
	**TEAEs n**	**Participants n (%)**	**TEAEs n**	**Participants n (%)**	**TEAEs n**	**Participants n (%)**	**TEAEs n**	**Participants n (%)**
GRADE 1	27	4 (100%)	27	4 (80%)	8	4 (80%)	24	5 (100%)
GRADE 2	9	4 (100%)	3	3 (60%)	2	2 (40%)	7	3 (60%)
GRADE 3	0	0 (0%)	0	0 (0%)	0	0 (0%)	0	0 (0%)
GRADE 4	0	0 (0%)	0	0 (0%)	0	0 (0%)	0	0 (0%)
GRADE 5	0	0 (0%)	0	0 (0%)	0	0 (0%)	0	0 (0%)
**TOTAL**	**36**	**4 (100%)**	**30**	**4 (80%)**	**10**	**4 (80%)**	**31**	**5 (100%)**

TEAE = Treatment Emergent Adverse Event; n = number

Grade 1 = Mild; Grade 2 = Moderate; Grade 3 = Severe; Grade 4 = Life-threatening; Grade 5 = Death

B: AG019/Teplizumab combination therapy cohorts (Phase 2a)

	**Adolescents, AG019/Teplizumab n=5**		**Adolescents, Placebo n=1**		**Adults, AG019/Teplizumab n=10**		**Adults, Placebo n=2**	
	**TEAEs n**	**Participants n (%)**	**TEAEs n**	**Participants n (%)**	**TEAEs n**	**Participants n (%)**	**TEAEs n**	**Participants n (%)**
GRADE 1	16	4 (80%)	2	1 (100%)	84	9 (90%)	27	2 (100%)
GRADE 2	9	5 (100%)	0	0 (0%)	35	8 (80%)	10	2 (100%)
GRADE 3	1	1 (20%)	0	0 (0%)	7	5 (50%)	0	0 (0%)
GRADE 4	0	0 (0%)	0	0 (0%)	1	1 (10%)	0	0 (0%)
GRADE 5	0	0 (0%)	0	0 (0%)	0	0 (0%)	0	0 (0%)
**TOTAL**	**26**	**5 (100%)**	**2**	**1 (100%)**	**127**	**10 (100%)**	**37**	**2 (100%)**

TEAE = Treatment Emergent Adverse Event; n = number

Grade 1 = Mild; Grade 2 = Moderate; Grade 3 = Severe; Grade 4 = Life-threatening; Grade 5 = Death

ESM Table 4. Summary of treatment emergent adverse events according to System Organ Class
A: AG019 monotherapy cohorts (Phase 1b).

	**Adolescents, low dose n=4**		**Adolescents, high dose n=5**		**Adults, low dose n=5**		**Adults, high dose n=5**	
	**TEAEs n**	**Participants n (%)**	**TEAEs n**	**Participants n (%)**	**TEAEs n**	**Participants n (%)**	**TEAEs n**	**Participants n (%)**
Blood and lymphatic system disorders	7	3 (75%)	3	2 (40%)	0	0 (0%)	2	1 (20%)
Gastrointestinal disorders	5	2 (50%)	5	2 (40%)	1	1 (20%)	4	2 (40%)
General disorders and administration site conditions	2	1 (25%)	1	1 (20%)	0	0 (0%)	1	1 (20%)
Immune system disorders	1	1 (25%)	0	0 (0%)	0	0 (0%)	1	1 (20%)
Infections and infestations	4	4 (100%)	6	3 (60%)	8	4 (80%)	7	3 (60%)
Injury, poisoning and procedural complications	2	1 (25%)	3	2 (40%)	0	0 (0%)	4	2 (40%)
Investigations	3	2 (50%)	4	1 (20%)	0	0 (0%)	5	2 (40%)
Metabolism and nutrition disorders	3	2 (50%)	0	0 (0%)	0	0 (0%)	0	0 (0%)
Musculoskeletal and connective tissue disorders	2	1 (25%)	3	2 (40%)	0	0 (0%)	0	0 (0%)
Nervous system disorders	1	1 (25%)	2	1 (20%)	0	0 (0%)	2	1 (20%)
Psychiatric disorders	1	1 (25%)	1	1 (20%)	0	0 (0%)	0	0 (0%)
Respiratory, thoracic and mediastinal disorders	2	1 (25%)	2	1 (20%)	0	0 (0%)	1	1 (20%)
Skin and subcutaneous tissue disorders	1	1 (25%)	0	0 (0%)	1	1 (20%)	4	3 (60%)

TEAE = Treatment Emergent Adverse Event; n = number

No TEAEs were detected in the following System Organ Class (SOC): Cardiac disorders, Eye disorders, Vascular disorders. One TEAE was reported for the following SOCs: Ear and labyrinth disorders, Reproductive system and breast disorders (in 1 adolescent treated with AG019 low dose).

B: AG019/Teplizumab combination therapy cohorts (Phase 2a).

	Adolescents, AG019/Teplizumab n=5		Adolescents, Placebo n=1		Adults, AG019/Teplizumab n=10		Adults, Placebo n=2	
	**TEAEs n**	**Participants n (%)**	**TEAEs n**	**Participants n (%)**	**TEAEs n**	**Participants n (%)**	**TEAEs n**	**Participants n (%)**
Blood and lymphatic system disorders	1	1 (20%)	0	0 (0%)	17	5 (50%)	2	2 (100%)
Gastrointestinal disorders	4	3 (60%)	0	0 (0%)	28	7 (70%)	3	2 (100%)
General disorders and administration site conditions	4	2 (40%)	0	0 (0%)	8	4 (40%)	7	2 (100%)
Infections and infestations	0	0 (0%)	0	0 (0%)	10	7 (70%)	2	1 (50%)
Injury, poisoning and procedural complications	1	1 (20%)	0	0 (0%)	3	3 (30%)	0	0 (0%)
Investigations	6	4 (80%)	0	0 (0%)	18	5 (50%)	0	0 (0%)
Musculoskeletal and connective tissue disorders	1	1 (20%)	0	0 (0%)	9	3 (30%)	4	2 (100%)
Nervous system disorders	3	1 (20%)	0	0 (0%)	9	5 (50%)	11	2 (100%)
Psychiatric disorders	4	1 (20%)	2	1 (100%)	1	1 (10%)	0	0 (0%)
Reproductive system and breast disorders	0	0 (0%)	0	0 (0%)	2	2 (20%)	0	0 (0%)
Respiratory, thoracic and mediastinal disorders	1	1 (20%)	0	0 (0%)	8	3 (30%)	5	1 (50%)
Skin and subcutaneous tissue disorders	1	1 (20%)	0	0 (0%)	10	6 (60%)	1	1 (50%)

TEAE = Treatment Emergent Adverse Event; n = number

One TEAE was reported for the following SOCs: Cardiac disorders, Eye disorders, Immune system disorders, Vascular disorders (in 1 adult treated with AG019/Teplizumab); Ear and labyrinth disorders, Metabolism and nutrition disorders (in 1 adult treated with placebo).

ESM Table 5. Overview of participants with incomplete teplizumab treatment.

Age Group	Number of infusions	Reason for teplizumab discontinuation
Adolescent	2/12	Hy's Law
Adult	8/12	Elevated ALT and AST
Adult	1/12	Neutropenia
Adult	2/12	Cytokine Release Syndrome
Adult	11/12	Elevated ALT

ALT = alanine transaminase, ASP = aspartate aminotransferase

ESM Table 6. Responder analysis at 6 months and at 12 months, expressed as a proportion and as a percentage per age and treatment group.

		AG019 Monotherapy		AG019/Teplizumab Combination Therapy		Placebo	
		6M	12M	6M	12M	6M	12M
Number of Responders	Adults % (proportion)	55% (5/9)	22% (2/9)	70% (7/10)	67% (6/9)	0% (0/2)	0% (0/1)
	Adolescents % (proportion)	28% (2/7)	16% (1/6)	100% (4/4)	100% (4/4)	0% (0/1)	-
	Total % (proportion)	44% (7/16)	20% (3/15)	78% (11/14)	77% (10/13)	0% (0/3)	0% (0/1)
Mean change from baseline in C-peptide mean 2-hour AUC in responders	Adults (n)	102% (5)	76% (2)	127% (7)	114% (6)	-	-
	Adolescents (n)	113% (2)	104% (1)	124% (4)	108% (4)	-	-
	Total (n)	105% (7)	84% (3)	126% (11)	112% (10)	-	-

Data are based on the PP. The 2-hour mean C-peptide AUC was used to identify treatment responders (28); a patient was classified as responder when the change from baseline was either non-negative or, if negative, represented a coefficient of variance (CV) was ≤ 9.7% (28, 29). M=month, n=number. AUC = area under the curve.

ESM Fig. 1: Study Design

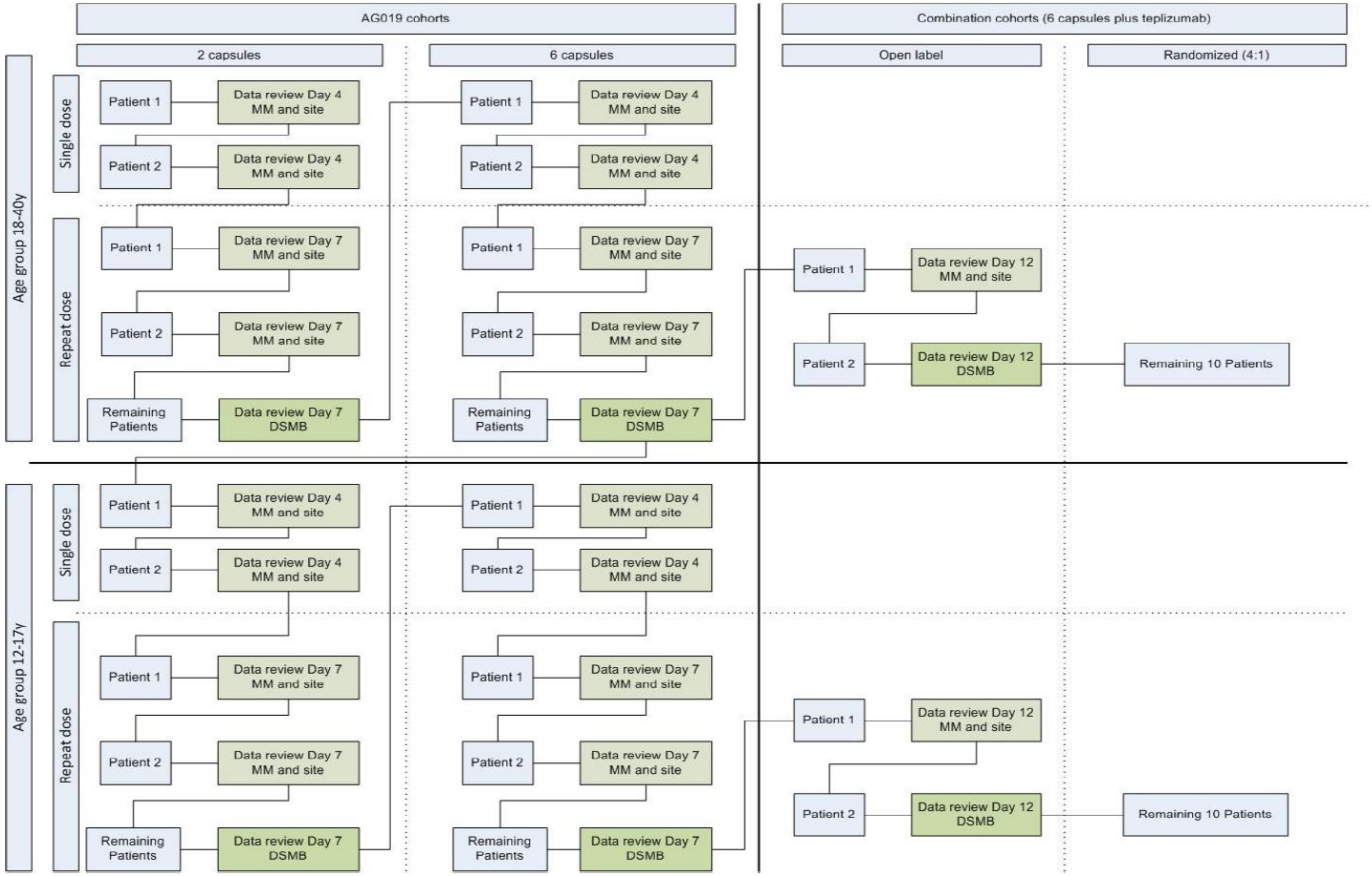

ESM Fig. 2. Gating strategy for antigen specific CD4 Tr1 (A) and Treg (B) and CD8 (C) T cell populations.

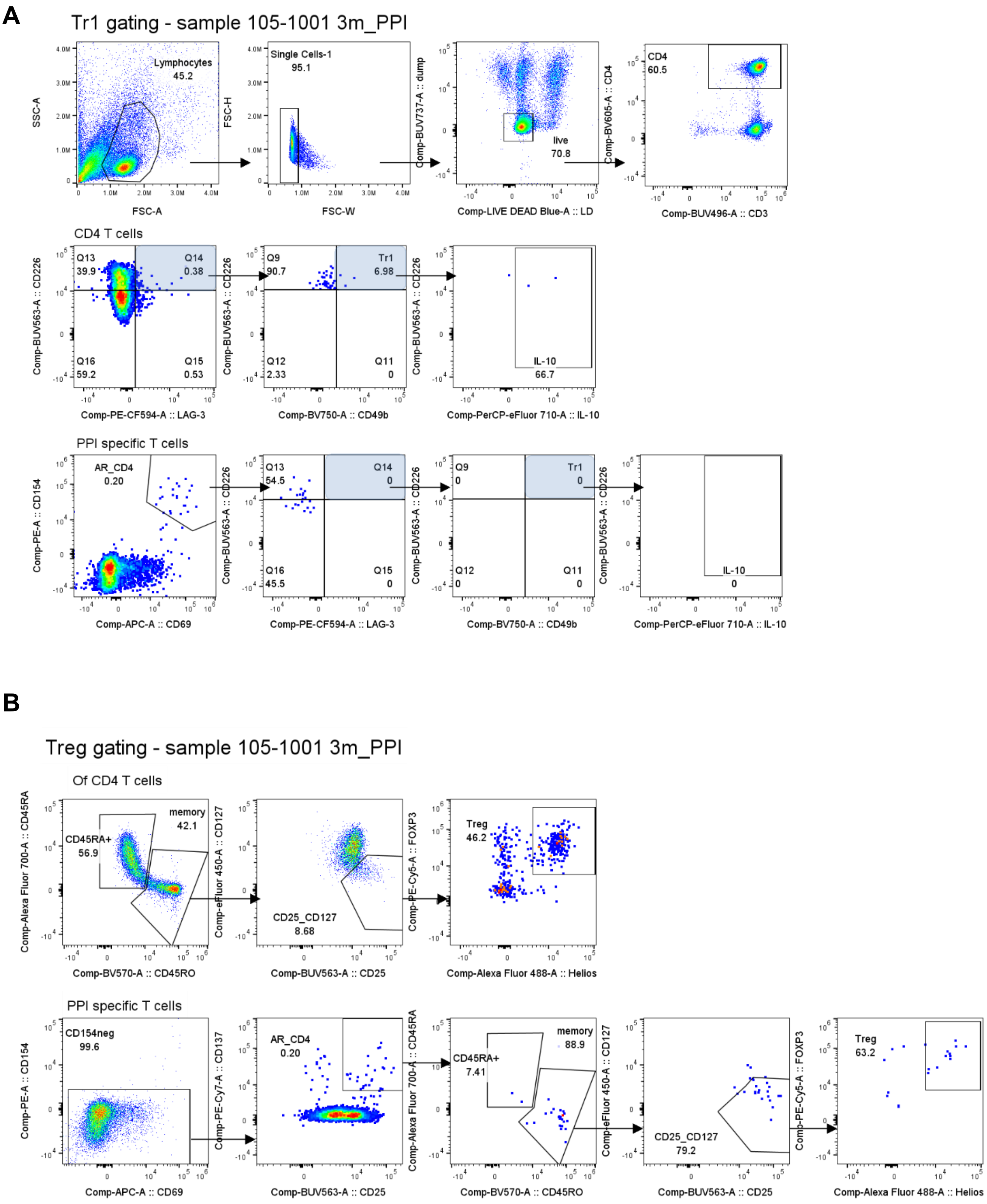

C CyTOF Boolean Tmr gating

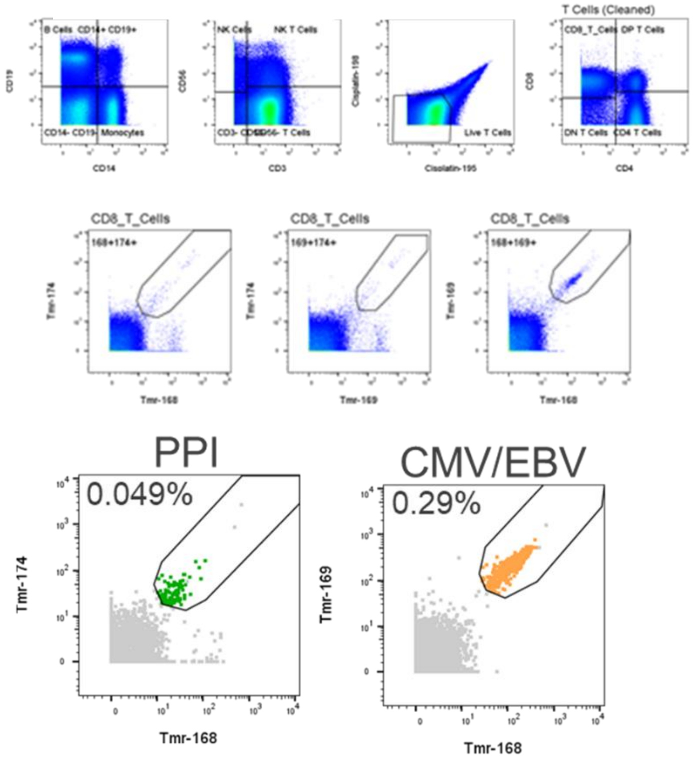

ESM Fig. 3: C-peptide Over Time in Patients Treated with AG019 Monotherapy, AG019/Teplizumab Combination Therapy and Placebo

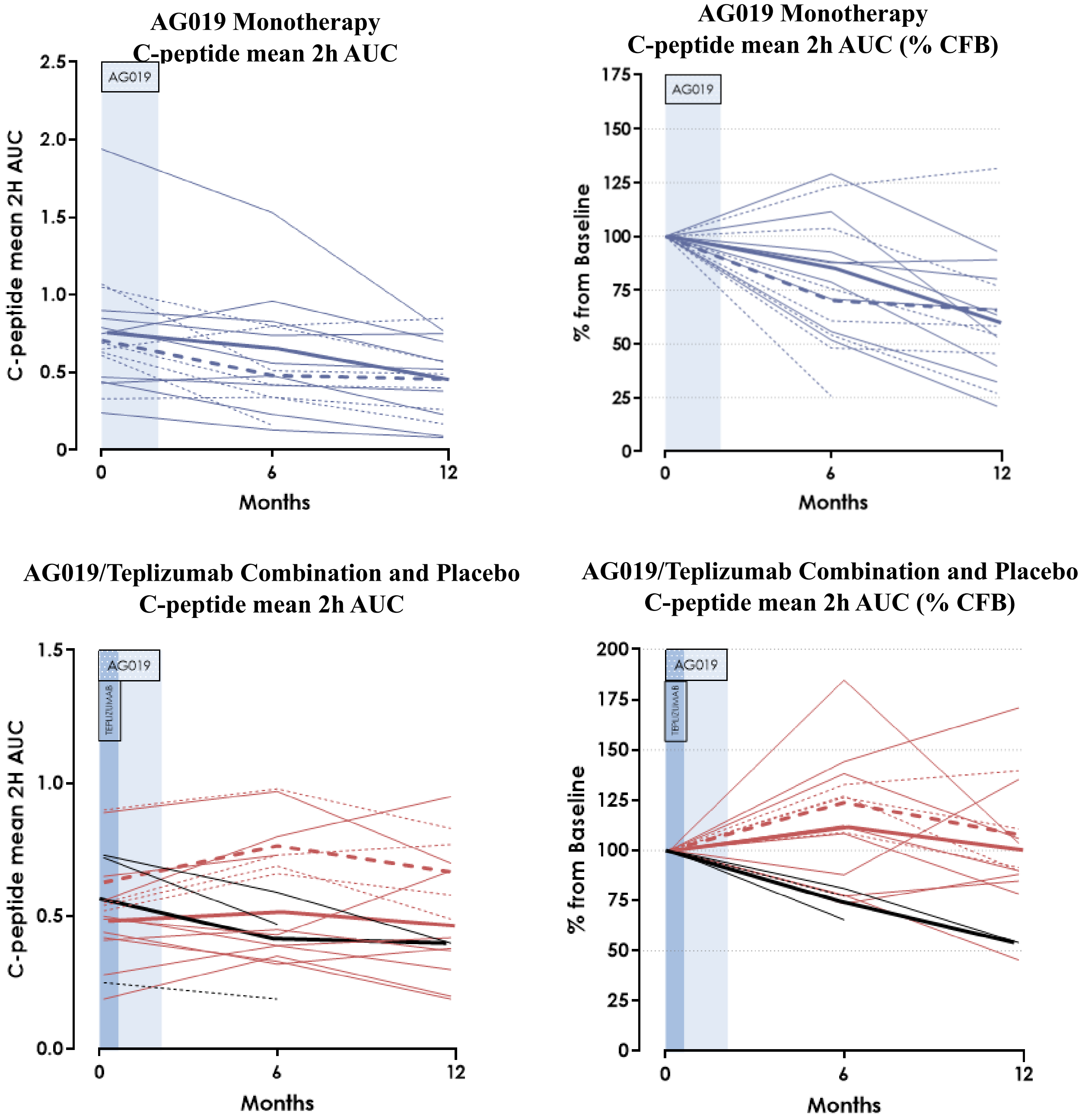

Data are based on the PP Analysis Set. Full lines represent individual (thin) and mean (thick) adult data, dotted lines represent individual (thin) and mean (thick) adolescent data and placebo data are indicated in black. CFB = change from baseline.

ESM Fig. 4. Mean change from baseline in C-peptide in Clinical Responders and Non-Responders at 6 months in AG019 Monotherapy and AG019/Teplizumab Combination Therapy.

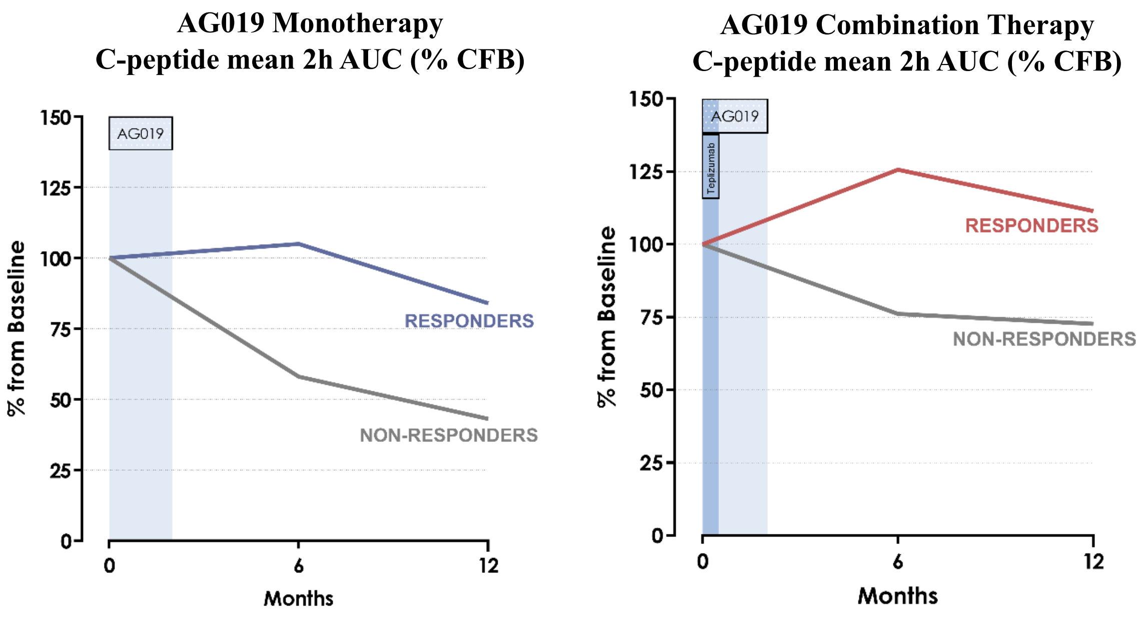

Data are based on a subset of the PP Analysis Set and are presented as mean values. AG019 monotherapy responders (n=7), AG019 monotherapy non-responders (n=9), AG019/Teplizumab combination therapy responders (n=11), AG019/Teplizumab combination therapy non-responders (n=3). CFB = change from baseline.
